# Association of fetal *FTO* gene variants with maternal postload glucose levels in pregnancy

**DOI:** 10.1038/s41366-025-01896-1

**Published:** 2025-08-28

**Authors:** Gábor Firneisz, Ákos Nádasdi, Botond A. Nemes, László Németh, Klara Rosta, Jürgen Harreiter, Alexandra Kautzky-Willer, Anikó Somogyi, Zoltán Benyó

**Affiliations:** 1 Institute of Translational Medicine, Semmelweis University, Budapest, Hungary; 2 Healthware Consulting Ltd., Budapest, Hungary; 3 Department of Obstetrics and Gynaecology, Medical University of Vienna, Vienna, Austria; 4 Department of Medicine III, Medical University of Vienna, Vienna, Austria; 5 Department of Medicine, Landesklinikum Scheibbs, Scheibbs, Austria; 6 Department of Internal Medicine and Haematology, Semmelweis University, Budapest, Hungary; 7 HUN-REN-SU Cerebrovascular and Neurocognitive Disease Research Group, Budapest, Hungary

**Keywords:** Obesity, Gestational diabetes, Development

## Abstract

**Objective:**

The *FTO* rs9939609 variant is a major common genetic risk factor of adult obesity. We hypothesized that the rs9939609 variant of the fetus alters the plasma glucose (PG) levels during oral glucose tolerance test (OGTT) routinely performed between the 24–28th gestational week.

**Methods:**

We analysed the data of mother-neonate pairs from our prior gestational diabetes mellitus (GDM) case-control study (Hungarian-Austrian set, *n* = 858) and the HAPO study European ancestry subset (HAPO-EUR, *n* = 1374) using pre-pregnancy body mass index (BMI) and maternal age as covariates. The rs8050136 (complete LD with rs9939609) was used in the HAPO-EUR data set.

**Results:**

Fetal *FTO* variants were associated (dominant genetic model) with decreased maternal 60’min PG values (ß_Hungarian-Austrian_ = −1.39 mmol/L, *p* = 1.97*10^−4^; ß_HAPO-EUR_ = −0.18 mmol/L, *p* = 4.36*10^−2^; ß_combined_ = −0.33 mmol/L, *p* = 2.11*10^−4^) and with reduced incremental area under glucose curve at OGTT (ß_Hungarian-Austrian_ =−1.70 mmol*h/L, *p* = 3.83*10^−4^; ß_HAPO-EUR_ = −0.23 mmol*h/L, *p* = 2.91*10^−2^; ß_combined_ = −0.39 mmol*h/L, *p* = 1.61*10^−4^).

**Conclusion:**

*FTO* risk variants carried by the fetus may indirectly influence maternal metabolism and could be associated with a flatter OGTT curve driven by the reduced 1 h postload PG levels in pregnancy.

## Introduction

Common variants of the *FTO* (alpha-ketoglutarate-dependent-dioxygenase) gene were found to be associated with obesity, visceral adiposity in adulthood, and strongly related traits including insulin resistance, type 2 diabetes mellitus (T2DM), and polycystic ovary syndrome (PCOS) [1–7]. The "*A*” risk allele of the rs9939609 variant was found to be associated with decreased weight loss and earlier weight regain after bariatric surgery [8], and also with decreased lean body mass and sarcopenia [9].

A recent meta-analysis reported that rs9939609 and rs8050136 variants of the *FTO* gene were significantly associated with T2DM worldwide [10]. The two best statistical indicators that quantify the extent of linkage disequilibrium (LD) indicate that the rs9939609 and rs8050136 variants are in complete LD in the European and US populations of European origin in the 1000 genomes database. Indication of complete LD implies full coinheritance [11].

Reportedly, both the maternal and infant “*AA”* genotype of rs9939609 may have a paradoxical effect in fetal life resulting in a higher risk of small for gestational age (SGA) neonate; however, this finding is inconsistent [12, 13]. Fetal homozygosity for the rs9939609 risk “*A*” allele was also associated with lower placental weight favoring the hypothesis that this gene variant may have a direct effect on the feto-placento-maternal unit during pregnancy. To our best knowledge, no previous study has reported any effect of fetal *FTO* gene variants on a maternal metabolic trait, such as plasma glucose levels in pregnancy.

Taking advantage of the genotype-phenotype database built during our prior gestational diabetes mellitus (GDM) case-control study, we aimed to assess the effect of the fetal rs9939609 gene variant on the fasting, 60’, and 120’ min plasma glucose (PG) levels during the routine oral glucose tolerance test (OGTT) at 24–28th gestational week (GW) in Hungarian-Austrian mother-neonate pairs [14].

To ensure the genotype-phenotype association found, we employed replication studies, which are widely recommended in genetic studies [15]. We replicated our study on the data obtained from independent mother-neonate pairs of European ancestry population subset from the Hyperglycemia and Adverse Pregnancy Outcome (HAPO) study [16].

## Subjects and methods

Genotype and phenotype data of mother-neonate duos (*n* = 858) were extracted from the Hungarian-Austrian database established in our prior GDM-genetic association study [14]. Data on the *FTO* rs9939609 and *MTNR1B* rs10830963 gene variants were used in this primary analysis in combination with relevant phenotype information on mothers and neonates. The 75 g OGTT was performed routinely between the 24th and 28th weeks of pregnancy in both countries; however, the 60 min PG data were available nearly exclusively from the Austrian cohort only due to differences between the national guidelines for the diagnosis of GDM.

In order to replicate our study on the data from the independent HAPO study [16] European ancestry subset, we applied for access to the phs000096/T2DMBIRTHWT database via the NIH dbGaP portal [17]. We followed the guidelines of the NCI-NHGRI working group on replication in association studies [15]. The *FTO* rs9939609 and *MTNR1B* rs10830963 gene variants were not directly represented on the Human610-Quad (Illumina) microarray used for genotyping of European ancestry participants in the HAPO study [16]. Therefore, we substituted the rs9939609 *FTO* variant with the rs8050136 gene variant in the replication analysis due to the complete linkage disequilibrium (LD: r^2^ = 1.0, D’ = 1.0) between the two variants based on the CEU (Utah residents with Northern and Western European ancestry) subpopulation data in Ensembl GRCh38 (1000 Genomes Project [18]).

PLINK software vs 1.9 was used to access the downloaded genotype data of the HAPO study and to select the appropriate *FTO* gene variant (rs8050136) on chromosome 16. The clinical data of European ancestry participants of the HAPO study were also extracted using the PLINK program, and the NIH data management and sharing policies (https://sharing.nih.gov/data-management-and-sharing-policy) were followed.

Subsequently, we combined the two datasets to assess the fetal *FTO* risk variant effect on maternal postload PG levels at OGTT where the risk variant represented the rs9939609 in Hungarian-Austrian and the rs8050136 in the HAPO dataset. There was no appropriate substitutional variant for the *MTNR1B* variant on the Human610-QUAD (Illumina) microarray, and as a consequence, the *MTNR1B* rs10830963 associated genetic effects could not be replicated in the HAPO dataset. Kruskal-Wallis tests, t-tests, and chi-squared tests were used to compare the maternal and neonatal clinical characteristics of the primary and replication study populations.

Linear regression models were applied to assess the effects of fetal *FTO* gene variants on maternal fasting, 60’ and 120’ min PG levels at 75 g OGTT. The distribution of the 120’ min PG values differed from normal therefore, a logarithmic transformation was applied to meet the assumptions of the linear regression. In addition to the fetal *FTO* risk genotypes, the pre-pregnancy BMI was used after logarithmic transformation [19, 20] (centralized) in combination with the maternal age (on 5 years scale, centralized) as covariates in model 1. Model 1 was extended in model 2 with maternal *MTNR1B* rs10830963 genotype identified as the strongest maternal genetic factor of GDM development in our prior study [14]. When the *FTO* and *MTNR1B* gene variants were used together, the same genetic model was applied for both variants. In addition, interaction analysis was performed between the maternal and fetal *FTO* gene variant effects and between the pre-pregnancy BMI and the fetal genetic effects.

Imputation was performed for missing pre-pregnancy BMI, maternal age, and 0’ min PG levels. For each subpopulation, a linear regression model was fitted to predict pre-pregnancy BMI based on age and 0’ min PG levels. These model predictions were then used to impute missing pre-pregnancy BMI values. Any missing age or 0’ min PG levels were imputed using mean values of appropriate study populations. After imputation, data from 125 participants were available for 60’ min PG level models and 654 for 120’ min models from Hungarian-Austrian population. In the HAPO population, the full cohort (1374 participants) was available for analysis after the imputation of both the 60’ and the 120’ min models.

In order to match the scales of the used variables, a logarithmic transformation and centralization by sample mean were applied for pre-pregnancy BMI, while maternal age was normalized using sample mean and variance.

Model diagnostics were performed using the DHARMa R package to assess the model fitting and to detect deviations from uniformity in y-direction on the residual vs. predicted quantile plots. Results of model diagnostics for non-extended dominant genetic models of 60’ PG values are indicated in Supplementary Fig. 1(A–C).

The incremental area under glucose curve at OGTT (AUC(gluc)INC) value was calculated based on the trapezoidal rule used for PG level per hour (AUC(gluc)INC = ((PG0 + PG60)/2 + (PG60 + PG120)/2)−PG0*2), where PGT stands for the PG value at minutes T) [21]. Even though we calculated hourly increases, we have retained the notations for minutes for consistency with the published formula. Effects of fetal *FTO* variants on the incremental area under glucose curve at OGTT were also modeled.

## Results

We could extract the *FTO* rs9939609 genotype and pregnancy-related phenotype data from 858 mother-neonate pairs from our preceding Hungarian-Austrian study [14]. Additionally, the data of 1374 independent mother-neonate pairs of European ancestry subset from the HAPO study were used in the replication cohort [17]. The summary of clinical data and allele frequency data in both the primary and replication cohorts is presented in Table 1. and Supplementary Table 1. Distribution of PG values in the original study and replication population is indicated in Supplementary Fig. 2.

**Table 1:** Maternal and offspring clinical characteristics in the primary (Hungarian-Austrian) and replication (HAPO-European ancestry) study populations.

	Hungarian-Austrian	HAPO-European	*p*
Mother neonate pairs (total n)	858	1374	
Maternal age at OGTT (mean, years [SD])	32.08 (5.46)	30.75 (5.26)	<0.001
Maternal pre-pregnancy BMI (mean, kg/m^2^ [SD])	24.64 (5.52)	24.57 (5.00)	0.78
0' min PG (mean, mmol/L [SD])	4.69 (0.63)	4.56 (0.38)	<0.001
60' min PG (mean, mmol/L [SD])*	8.43 (2.25)	7.34 (1.62)	<0.001
120' min PG (mean, mmol/L [SD])	6.56 (1.89)	6.07 (1.21)	<0.001
Pregnancy length (mean, weeks [SD])	39.11 (1.38)	39.94 (1.17)	<0.001
Offspring sex (*n*_male_ [%])	447 (54.6)	682 (49.6)	0.026
SGA (*n* [*n*/n, %])	28 (3.9)	124 (9.0)	<0.001
AGA (*n* [*n*/*n*, %])	559 (78.6)	1017 (74.0)	0.023
LGA (*n* [*n*/*n*, %])	124 (17.4)	233 (17.0)	0.829

Neonates whose weight was < the 10th percentile or > the 90th percentile (obtained from regional databases) for gestational age were classified as small for gestational age or large for gestational age, respectively; *p*-values were calculated using t- or chi-squared tests (for continous and for categorical variables, respectively).

*SGA* small for gestational age, *AGA* appropriate for gestational age, *LGA* large for gestational age.

* 60' min PG values at OGTT were routinely only measured at the Austrian study population and in theHAPO study participants, but was not recommended in Hungary.

### Fetal FTO rs9939609 associated effects on maternal plasma glucose levels at 75 g OGTT

**Fig. 1:**
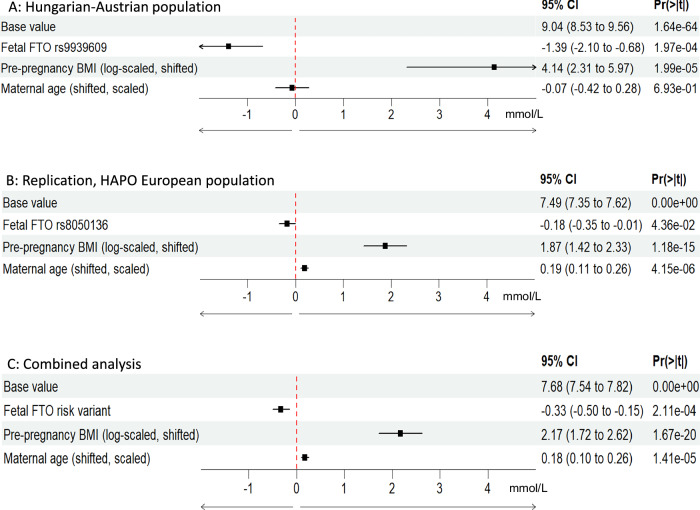
Dominant genetic models of fetal *FTO* risk variants with maternal age and BMI covariates for the 60’ min maternal plasma glucose values during OGTT in 24–28th gestational weeks. **A** Primary analysis in Hungarian-Austrian study (*n* = 125). **B** Replication analysis in the HAPO-European dataset (*n* = 1374). **C** Analysis in the combined dataset (*n* = 1499). Genotype data of rs9939609 were only available in the primary analysis. The rs8050136 (in complete linkage disequilibrium with rs9939609 in European descent populations) was used as a substitutional variant in the replication analysis.

Remarkably, the fetal *FTO* rs9939609 variant was associated with significant reductions of postload PG levels using pre-pregnancy BMI (log scaled [19, 20]) and maternal age as covariates, with the highest genetic effect size in the 60’ min PG (dominant model [/carrying the “*A*” allele]: −1.39 mmol/L, 95% CI:−2.1 to −0.68, *p* = 1.97*10^−4^ Fig. 1A; additive model [/“*A*” allele]: −1.21 mmol/L, 95% CI:−1.70 to −0.72, *p* = 3.74 × 10^−6^) in the primary analysis. We also analyzed maternal PG levels at 120 min, but due to their distribution, we used a logarithmic scale. In this case, the genetic effects obtained can be transformed into multiplicative effects (by taking the exponent of the coefficients). Although this is not directly comparable to the results of the 60 min PG model, the effects appear to be smaller (dominant model [/ carrying the “*A*” allele]: 94.17%, 95% CI: 89.58–98.02%, *p* = 9.00 × 10^−3^; additive model [/“*A*” allele]: 94,17%, 95% CI: 91.4–97.04%, *p* = 2.53 × 10^−4^).

Subsequently, we extended the highest genetic effect size (dominant) model for the 60’ min PG with the rs10830963 *MTNR1B* gene variant (the maternal gene variant with highest effect size on PG levels in our prior study [14] as a cofactor). The expected PG increasing effect was detectable for the maternal carrying of the rs10830963 *MTNR1B* variant (/carrying the “*G*” risk allele: 0.87 mmol/L, 95% CI: 0.08–1.65, *p* = 3.2*10^−2^—genotype data from the Hungarian-Austrian database), and this did not alter the glucose lowering effect of the fetal *FTO* gene variant on the 60’ min maternal PG values (/carrying the “*A*” allele: −1.39 mmol/L, 95% CI:−2.2 to −0.62, *p* = 6.1*10^−4^) when analyzed together in the model.

### Replication results on European ancestry subset of the HAPO study and its combination with the Hungarian-Austrian dataset

The effect of the rs8050136 *FTO* gene variant was assessed in the replication dataset to substitute the rs9939609 *FTO* variant that was not directly available for HAPO-EUR dataset. The results of the replication analysis also indicated that the genetic effect of fetal rs8050136 *FTO* gene variant was present on the 60’ min PG values (under the dominant model) in the HAPO European subset (−0.18 mmol/l, 95% CI: −0.35 to 0.01, *p* = 4.36 × 10^−2^) (Fig. 1B).

When the HAPO European ancestry subset and the Hungarian-Austrian datasets were merged, the combined effect of the *FTO* risk gene variants (rs8050136 and rs9939609) was also associated with decreased 60’ min PG values at OGTT (-0.33 mmol/l, 95% CI: −0.50 to −0.15, *p* = 2.11 × 10^−4^) under the dominant model (Fig. 1C).

### Consistency of the fetal genetic effect on 60’ min PG value across the pre-pregnancy BMI spectrum

**Fig. 2:**
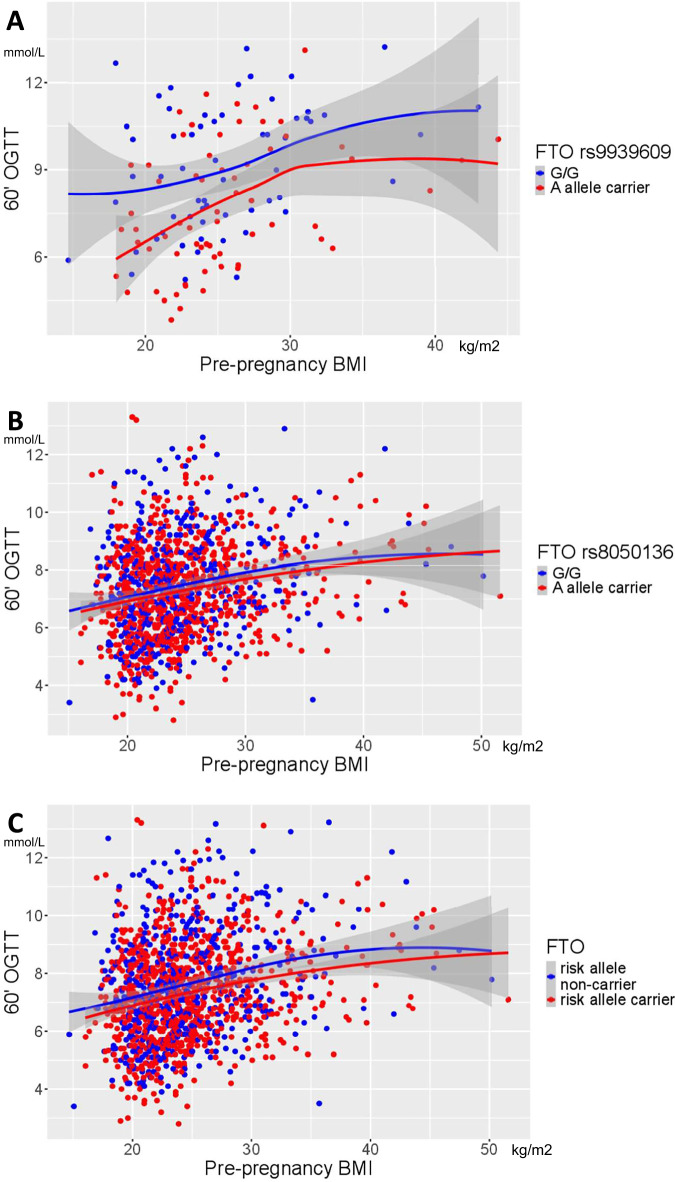
Prediction of maternal 60’ plasma glucose values at OGTT during pregnancy stratified by fetal *FTO* variant risk allele carrying over the pre-pregnancy BMI spectrum. **A** Hungarian-Austrian study population. **B** HAPO-European study population. **C** Combined populations.

The prediction of the 60’ min PG level under the dominant genetic model of fetal *FTO* variant indicated a constant fetal *FTO* risk variant associated genetic effect across the entire pre-pregnancy BMI spectrum (Fig. 2). This is consistent with the finding that there is no interaction between the fetal *FTO* risk variant associated genetic effect and the pre-pregnancy BMI effect on the 60’min PG value at OGTT (Supplementary Fig. 3).

### Fetal FTO risk variant associated effects on incremental area under glucose curve at OGTT (AUC(gluc)INC)

**Fig. 3:**
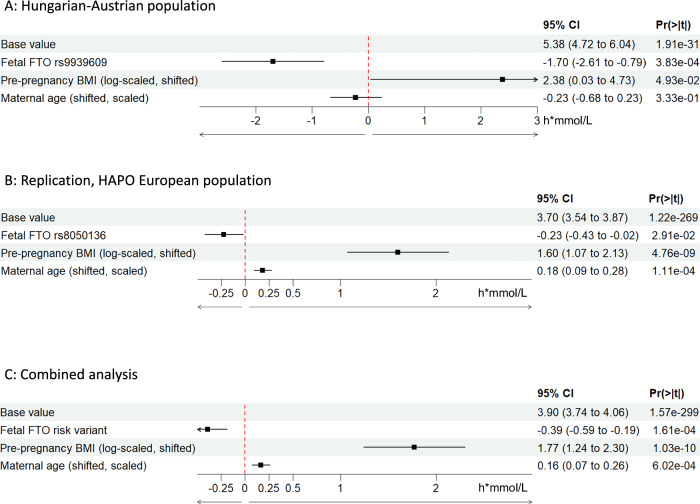
Effect of fetal *FTO* risk variants (dominant genetic model) on maternal incremental area under glucose curves (AUC_(gluc)_INC) at OGTT during pregnancy. **A** Hungarian-Austrian study population (*n* = 125). **B** HAPO-European study population (*n* = 1374). **C** combined populations (*n* = 1499). The genotype data of rs9939609 were available only in the primary (Hungarian-Austrian) analysis and the rs8050136 was used as a substitutional variant in the HAPO-European subset.

We found lower AUC(gluc)INC values were associated to the fetal *FTO* risk variants under the dominant genetic model both in our primary study and in the replication analysis on the European ancestry subset of the HAPO study, as well as when the two datasets were combined (Fig. 3A–C, respectively). This indicates that flatter OGTT curves are associated with the fetal *FTO* risk variants.

### Analysis of fetal-maternal genetic interaction on 60’ min PG value and AUC(gluc)INC

The maternal *FTO* genotype had neither an effect on the maternal 60’ min PG nor on the AUC(gluc)INC during OGTT in any of the populations assessed (Hungarian-Austrian, HAPO-EUR, Combined). This also did not alter the fetal effect (Supplementary Figs. 4 and 5.). The fetal-maternal interaction components of the *FTO* gene variant associated effects were neither significant on the 60’ min PG nor on the AUC(gluc)INC during OGTT in the subsequently performed interaction analysis. The fetal genetic effect sizes remained similar, but with the extension of the models, its significance levels were weakened (Supplementary Figs. 6 and 7).

## Discussion

To our best knowledge, we first report that a common fetal gene variant affects maternal metabolism, specifically that *FTO* risk gene variants are associated with reduced maternal postload PG values during routine OGTT between the 24th–28th weeks of pregnancy.

Our primary analysis based on the genotype-phenotype data of 858 mother-neonate pairs from our prior Hungarian-Austrian GDM-genetic study [14] indicated that a common intron variant (rs9939609) in the *FTO* gene was associated with decreased 60’ and 120’ min maternal PG levels. This fetal genetic effect on maternal glucose utilization was present after adjustments to maternal pre-pregnancy BMI and age at OGTT as the two major clinical risk factors for GDM development. This significant fetal genetic effect was detected even after we extended the model with maternal *FTO* risk gene variant that had no effect on postload PG levels and also after the inclusion of *MTNR1B* rs10830963 genotype to the model, due to that this latter variant is the maternal genetic factor most robustly associated with GDM development and antenatal insulin therapy initiation [14, 22].

We replicated our association analysis on a dataset obtained from the European ancestry mother–infant pairs of the HAPO study that was entirely independent with a similar pregnant study population [16]. In the replication study, we used a substitutional *FTO* gene variant (rs8050136) that was in complete LD with rs9939609 in populations of European origin. This enabled us to assess the effect of the fetal *FTO* risk variant on maternal postload PG levels in an additional 1374 mother-neonate duos and with the HAPO and Hungarian-Austrian datasets combined.

Remarkably, the highest genetic effect size of the fetal rs9939609 *FTO* variant found on the 60’ min PG values among the OGTT time points in our Hungarian-Austrian dataset may deserve clinical interest with a mean decrease of −1.39 mmol/L (dominant genetic model). The significance of the 1 h PG at OGTT in pregnancy was explored by Di Cianni et al. who found lower insulin secretion–sensitivity index (ISSI, a measure of beta cell response) in pregnant women who had only 1 h PG above the threshold when compared to those with increased fasting PG only or increased late (2 h or 3 h) PG values only during a 100 g OGTT [23, 24]. A number of placental hormones are physiologically elevating maternal glucose levels in order to maintain sufficient maternal glycemia for placental glucose transfer meeting the fetal nutrition needs in normal pregnancies [25–27]. Therefore, one may imagine that the novel observation presented here might be explained by the “diabetogenic” placental effects (that are otherwise also part of the normal maternal glucose regulation in pregnancy and compromise the 1st phase insulin response) that could be reduced in mothers who carry fetuses with the *FTO* risk variant. This results in the lower 1 h PG values and flatter curve at OGTT. In theory, *FTO* gene variants might interact with the maternal fuel supply [28].

Associations were reported in GDM focused studies between increased 1-h PG at OGTT in pregnancy and elevated cord insulin/C-peptide levels and increased prevalence of neonates with higher birthweight percentiles [29, 30]. Consistently, it could also be hypothesized that lower 1-h PG levels might be associated with lower birthweight percentiles, but this is less well studied [30]. It was also reported that the 1-hour postprandial and the overall mean blood glucose levels were the most closely correlated parameters with fetal growth. The excessively tight glycemic control was also associated with increased incidence of SGA neonates [31].

A recent retrospective analysis of a total of nearly half a million women with 2 singleton pregnancies found that 23% of mothers with GDM at any pregnancy had presented with GDM in both pregnancies, and 30.9% of them had GDM only in their first pregnancy, but not in the 2nd when they were older [32]. While we agree with the explanation suggested by the authors that GDM in the 1st pregnancy may increase the motivation for lifestyle change and better eating habits resulting in GDM risk reduction for the 2nd pregnancy, this assumption remains unsupported by firm data on inter-pregnancy BMI and the during pregnancy weight gain changes in their specific cohort [32]. In contrast, other data indicated that 96.9% of women with obesity prior to pregnancy remained with obesity at 1 year postpartum [33]. This data on overwhelmingly unsuccessful weight management in the 1st postpregnancy year theoretically may be due to other factors, such as genetic diversity of different fetuses in the same mother and the related variation of the feto-placental unit between pregnancies. These factors may contribute to the explanation for the above findings however, they are not restricted exclusively to the changes in classical maternal clinical risk factors. Although both fetal and maternal genetic factors were described to influence the choice of anti-hyperglycemic therapy during pregnancy in *GCK*-MODY patients (e.g.: no treatment is recommended if the fetus is suspected to have inherited *GCK*-MODY) [34]—to our best knowledge—there are no prior reports suggesting that a fetal gene variant might have an effect on maternal glycemia during OGTT in pregnancy.

Despite our preliminary assessment that the HAPO database European ancestry subset could be highly similar to our database, we detected differences after we were granted access to the actual HAPO data. Although no differences were detected in the pre-pregnancy BMI values and the minor allele frequencies (MAFs) and fetal genotype distributions of the *FTO* gene variants assessed, our study population was a case-control study population enriched with GDM mothers with differing maternal age. Remarkably, this resulted in lower PG values in the HAPO study population at all OGTT time points compared to our prior GDM case control study. The mean difference of PG levels at 1 h between the two studies exceeded 1 mmol/L, and the 60’ min PG value was our primary outcome of interest in this analysis. Consistently, the proportion of SGA neonates was halved in our Hungarian-Austrian study compared the HAPO study. These differences between the primary and the replication analyses in combination were that only a substitutional *FTO* gene variant effect was available for the HAPO European ancestry dataset which might explain the still significant, but smaller genetic effect associated to the *FTO* risk variant when the HAPO replication set was analyzed separately.

Although a number of mechanisms have been proposed [35–38] to explain the association between *FTO* risk gene variants and adult obesity [1, 3, 39], a recent meta-regression analysis reported that the association between the rs9939609 *FTO* gene variant and BMI largely varies with age [40]. There is an inverse association between the *FTO* risk variant with BMI from infancy to 3 years of age [41] that later turns to the opposite and a positive association occurs at ages 5.5 to 13 years and later [40]. Consistently, a study on Finnish children reported that the rs9939609 gene variant’s BMI increasing effect only becomes evident after 7 years of age [42]. Descamps et al. reported that the rs9939609 variant was associated with low birthweight and suggested a paradoxical effect for risk *FTO* gene variants in fetal life [43]. It was subsequently found that both maternal and neonatal “*A/A*” rs9939609 risk genotypes were associated with a higher risk of small for gestational age and spontaneous preterm delivery [12]. It was also raised that lower birth weight makes individuals more prone to a higher adult BMI in an *FTO* risk genotype dependent manner [44]. In addition, a flat OGTT curve is reportedly associated with higher SGA rates and lower birth weight [45] and in independent publications higher SGA rate and lower birth weight were also associated with the rs9939609 *FTO* variant [12, 43]. In addition, lower mean upper arm circumference (MUAC) scores were described in rs9939609 *A* allele carrier neonates born to *A* allele carrier mothers [46] that are typically indicative for lower lean body mass [47].

A positive relationship was found between placental *FTO* mRNA expression and fetal weight suggesting that *FTO* gene variants might be implicated in a placental development and function in animal experiments [48]. It may be raised that the lower placental weight associated to the infant *FTO* rs9939609 “*AA*” homozygosity [12] might result in intrauterine conditions somewhat similar to that of a deficient maternal nutritional and calorie status [49] that could lead to a fetal reprogramming eventually resulting in increased added sugar intake after birth as observed already in healthy young adults carrying the *FTO* risk “ *A*” allele [50].

## Conclusion

In summary, here, to our best knowledge, we first present results suggesting that a fetal gene variant has an effect on maternal metabolism during pregnancy. Specifically, we first report a flatter OGTT curve driven by the decreased 60’ min postload PG levels during OGTT in pregnancy in those mothers who carry fetuses with the *FTO* rs9939609/rs8050136 risk alleles that are major common genetic risk factors of obesity development later in life. Although this report already comprises a replication on an independent set of mother-neonate pairs, additional replication studies might still be needed to confirm this novel phenomenon. The role of fetal *FTO* risk variants in the regulation of maternal glucose metabolism during pregnancy and its subsequent contribution to obesity risk in adulthood needs to be further explored.

## Supplementary information

Supplementary Materials

## Data Availability

Anonymized participant data for the Hungarian-Austrian dataset might be available for non-commercial purposes upon data sharing requests directed to the corresponding author. Proposals will be reviewed and approved by the Semmelweis University, Hungary and the Medical University of Vienna, Austria. The investigators are assessed on the basis of scientific merit and potential conflict of interest and such requests also have to be approved at institutional level. After approval of a proposal, data can be shared through a secure online platform after signing a data access agreement. NIHeRA commons account holders may submit request to access the HAPO data through the NIH database of Genotypes and Phenotypes (dbGaP) for controlled access as described: https://www.ncbi.nlm.nih.gov/books/NBK570242/.
