# Supplementary material for: Association of fetal FTO gene variants with maternal postload glucose levels in pregnancy
Source: Int J Obes (Lond). 2025 Aug 28;49(11):2338–45. doi: 10.1038/s41366-025-01896-1 (PMC12583136; doi:10.1038/s41366-025-01896-1)
Supplement: Supplementary file 1 — Supplementary Materials [file 41366_2025_1896_MOESM1_ESM.pdf]

Supplementary Figures and Table to Association of fetal *FTO* gene variants with maternal postload glucose levels in pregnancy

Gábor Firneisz[1*]**,** *Ákos Nádasdi*[1], *Botond A. Nemes*[1], *László Németh*[1,2], *Klara Rosta*[3], *Jürgen Harreiter*[4,5] *Alexandra Kautzky-Willer*[4], *Anikó Somogyi*[6], *Zoltán Benyó*[1,7]

[1]Institute of Translational Medicine, Semmelweis University, Budapest, Hungary

[2]Healthware Consulting Ltd., Budapest, Hungary

[3]Department of Obstetrics and Gynaecology, Medical University of Vienna, Vienna, Austria

[4]Department of Medicine III, Medical University of Vienna, Vienna, Austria

[5]Department of Medicine, Landesklinikum Scheibbs, Scheibbs, Austria

[6]Department of Internal Medicine and Haematology, Semmelweis University, Budapest, Hungary

[7]HUN-REN-SU Cerebrovascular and Neurocognitive Disease Research Group, Budapest, Hungary

Contact info: *Gábor Firneisz, M.D., Ph.D.*

Institute of Translational Medicine, Semmelweis University

1094 Budapest, Tűzoltó Street 37-49, Hungary

Email: firneisz.gabor@semmelweis.hu

Supplementary files

Supplementary Figure 1. Model diagnostics for dominant genetic models for 60' plasma glucose values during OGTT 1

Supplementary Figure 2. Distribution of plasma glucose (PG) values at 75g oral glucose tolerance test (OGTT) in the Austro-Hungarian GDM case-control study population and the HAPO European ancestry study subpopulation 2

Supplementary Figure 3. Interaction analysis of fetal *FTO* gene variant and maternal pre-pregnancy BMI effects on 60' plasma glucose values (during OGTT) in Hungarian-Austrian (A), HAPO_EUR (B) and in the combined study population (C) 3

Supplementary Figure 4. Fetal and maternal *FTO* genetic effect on 60' plasma glucose values (during OGTT) in Hungarian-Austrian (A), HAPO (B) and in the combined population (C) 4

Supplementary Figure 5. Fetal and maternal *FTO* genetic effect on AUC(gluc)INC during OGTT in Hungarian-Austrian (A), HAPO_EUR (B) and in the combined study populations (C) AUC(gluc)INC, incremental area under glucose curve 5

Supplementary Figure 6. Interaction analysis of fetal and maternal *FTO* genetic effects on 60' plasma glucose values (during OGTT) in Hungarian-Austrian (A), HAPO (B) and in the combined population (C) 6

Supplementary Figure 7. Interaction analysis of fetal and maternal *FTO* genetic effects on AUC(gluc)INC during OGTT in Hungarian-Austrian (A), HAPO_EUR (B) and in the combined study populations (C) AUC(gluc)INC, incremental area under glucose curve . 7

Supplementary Table 1. Case numbers and genotype distributions of the *FTO* risk gene variants in the primary (Hungarian-Austrian, panel "A") and replication (HAPO-EUR) study (panel "B") populations 8

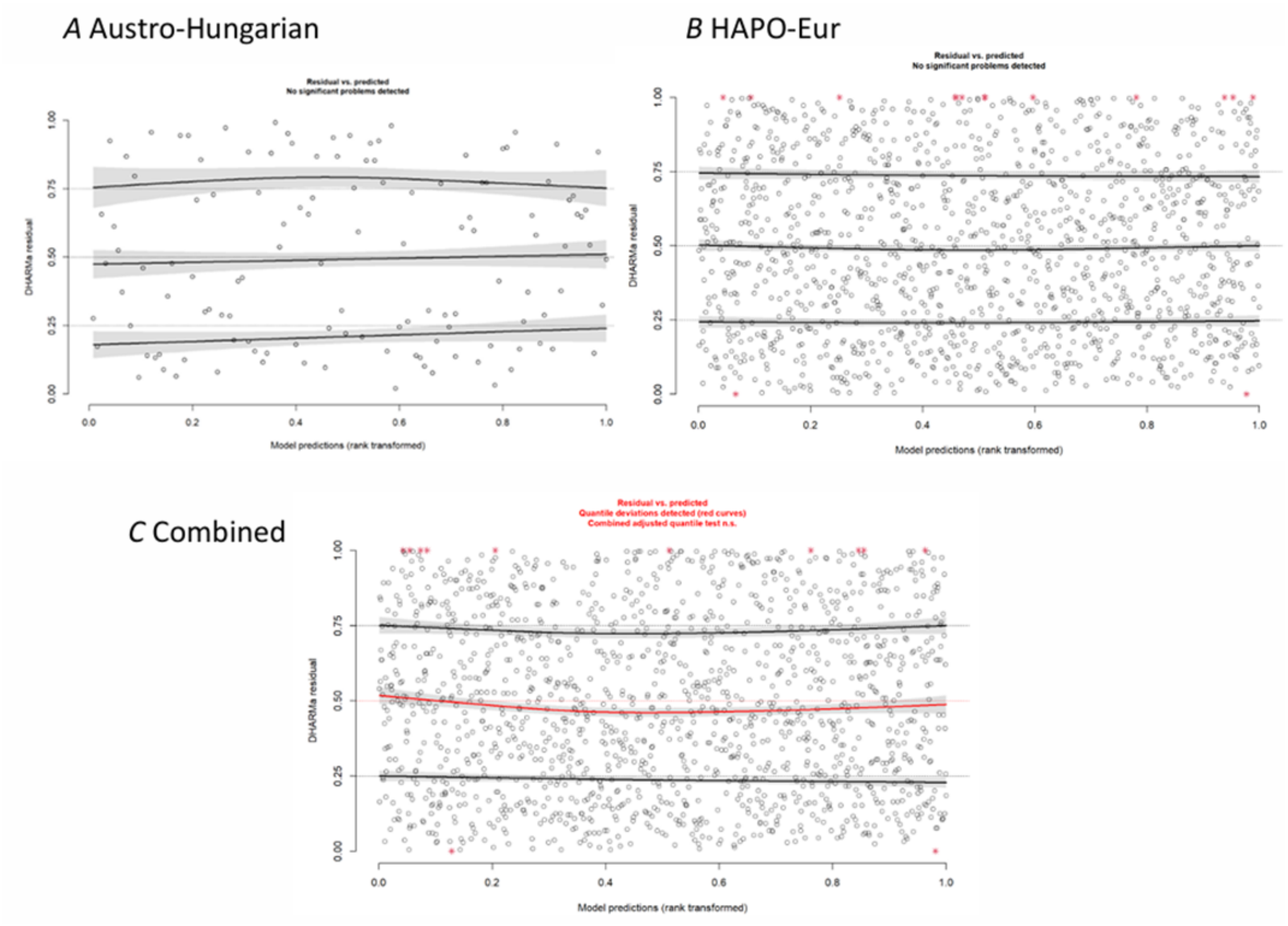

Supplementary Figure 1. Model diagnostics for dominant genetic models for 60’ plasma glucose values during OGTT

A: Hungarian-Austrian (n=125)

B: HAPO-European (n=1374)

C: Combined study populations (n=1499)

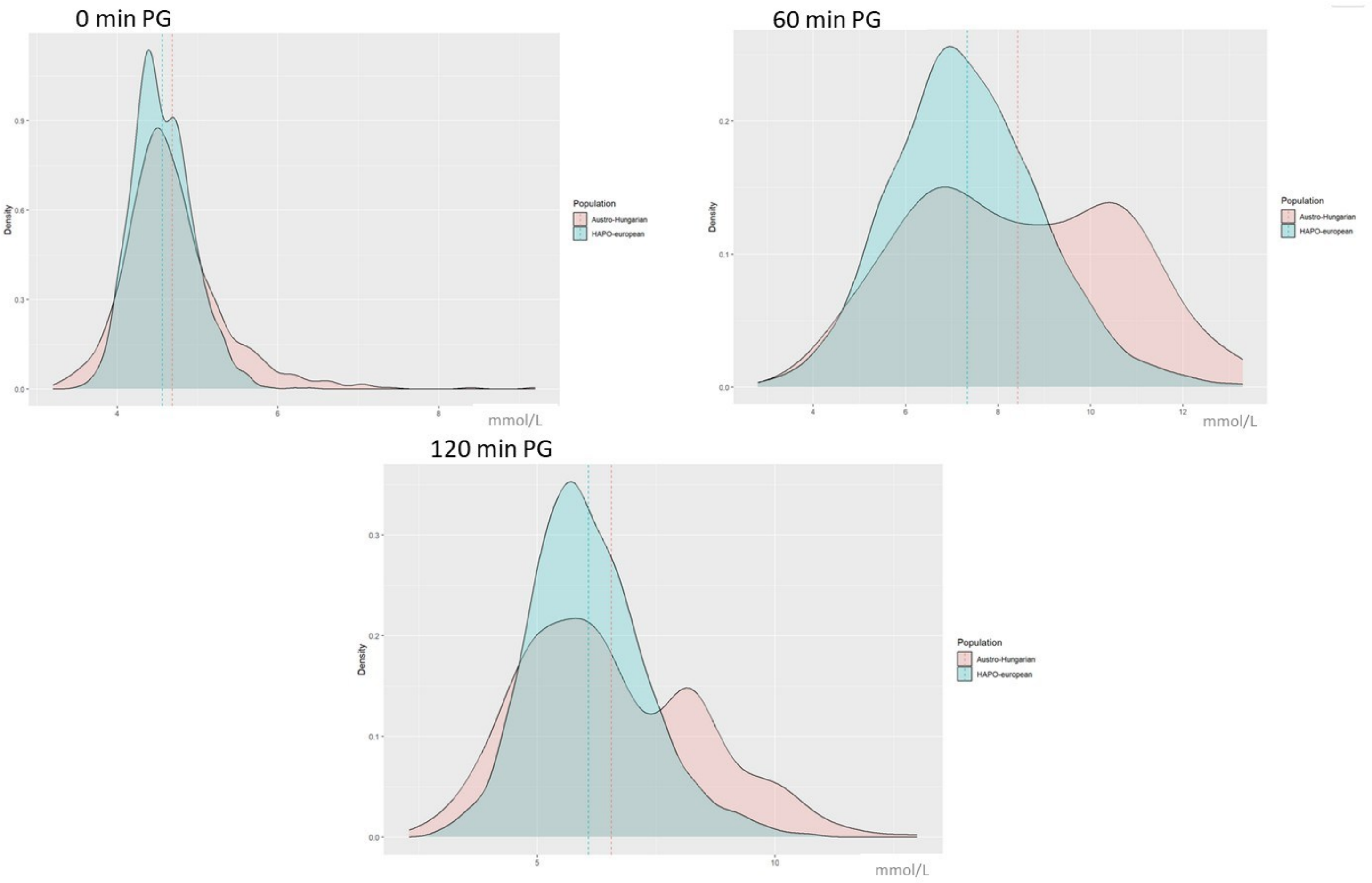

Supplementary Figure 2. Distribution of plasma glucose (PG) values at 75g oral glucose tolerance test (OGTT) in the Austro-Hungarian GDM case-control study population and the HAPO European ancestry study subpopulation

Fasting, 60’ min and 120’ min PG values respectively; GDM, Gestational Diabetes Mellitus; HAPO, Hyperglycemia Adverse Pregnancy Outcome study

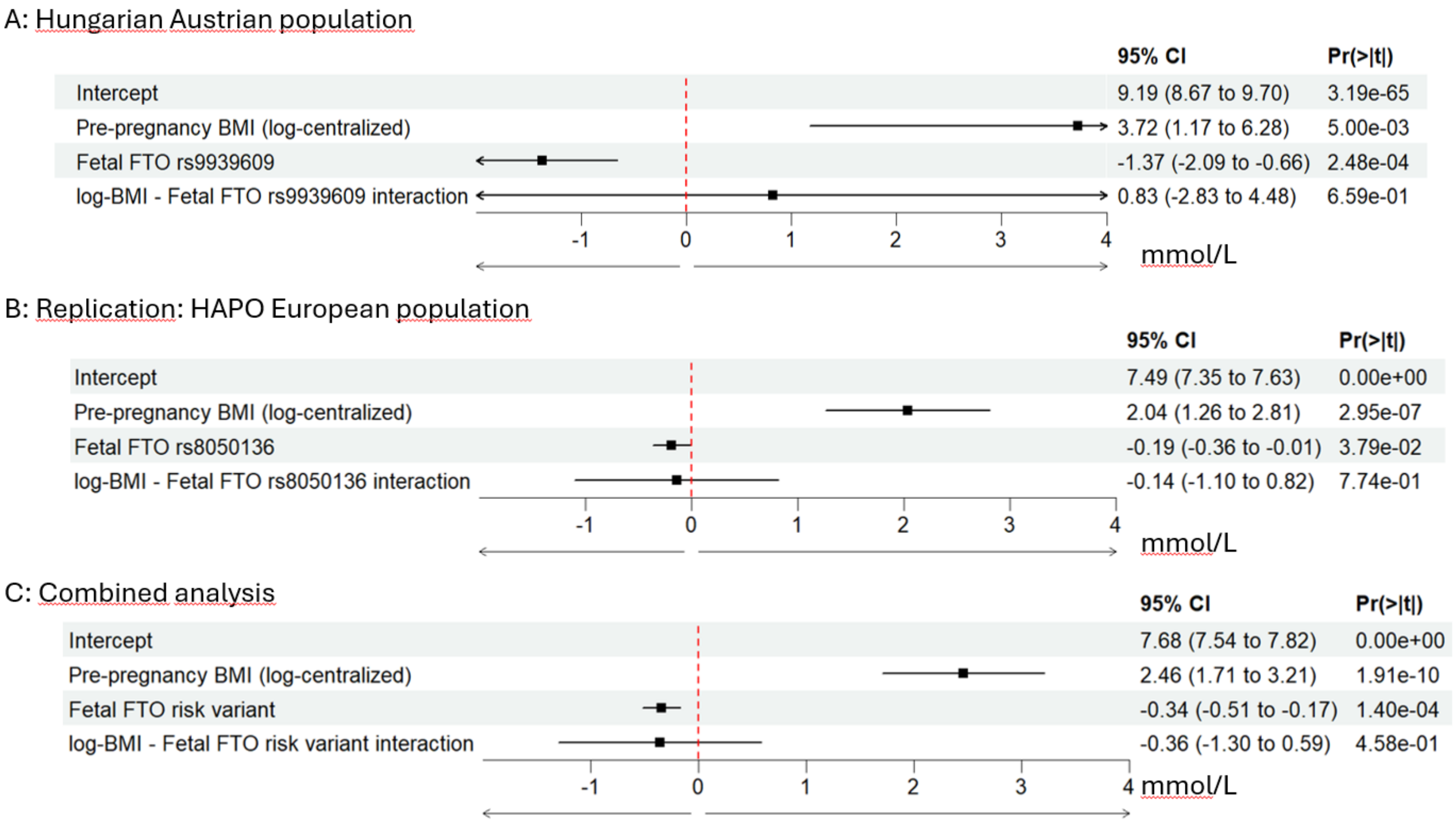

Supplementary Figure 3. Interaction analysis of fetal *FTO* gene variant and maternal pre-pregnancy BMI effects on 60' plasma glucose values (during OGTT) in Hungarian-Austrian (A), HAPO_EUR (B) and in the combined study population (C)

HAPO, Hyperglycemia Adverse Pregnancy Outcome study; HAPO-EUR, Hyperglycemia Adverse Pregnancy Outcome study subpopulation of European ethnic origin; HUN-AUS; Hungarian-Austrian

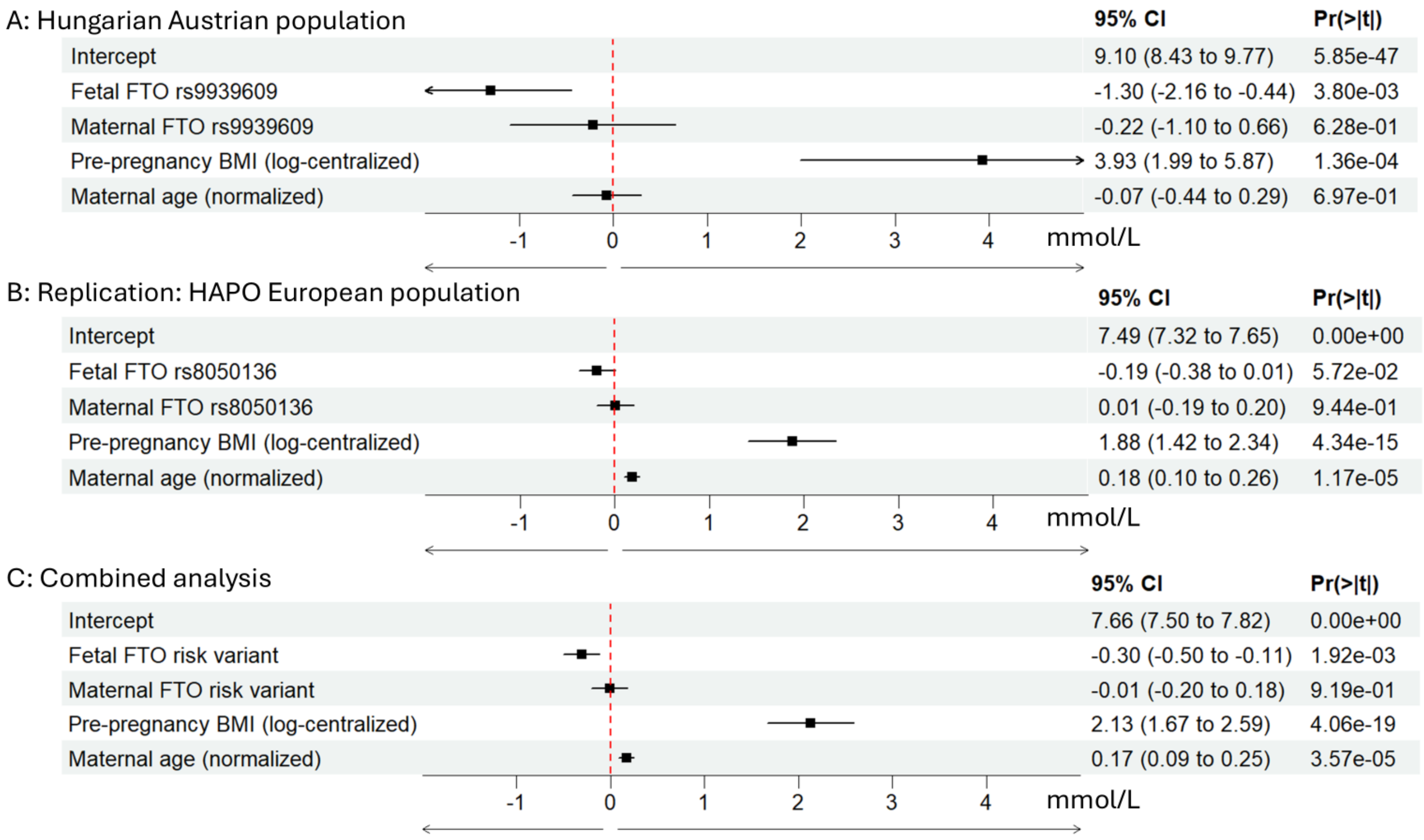

Supplementary Figure 4. Fetal and maternal *FTO* genetic effect on 60' plasma glucose values (during OGTT) in Hungarian-Austrian (A), HAPO-EUR (B) and in the combined population (C)

HAPO-EUR, Hyperglycemia Adverse Pregnancy Outcome study subpopulation of European ethnic origin

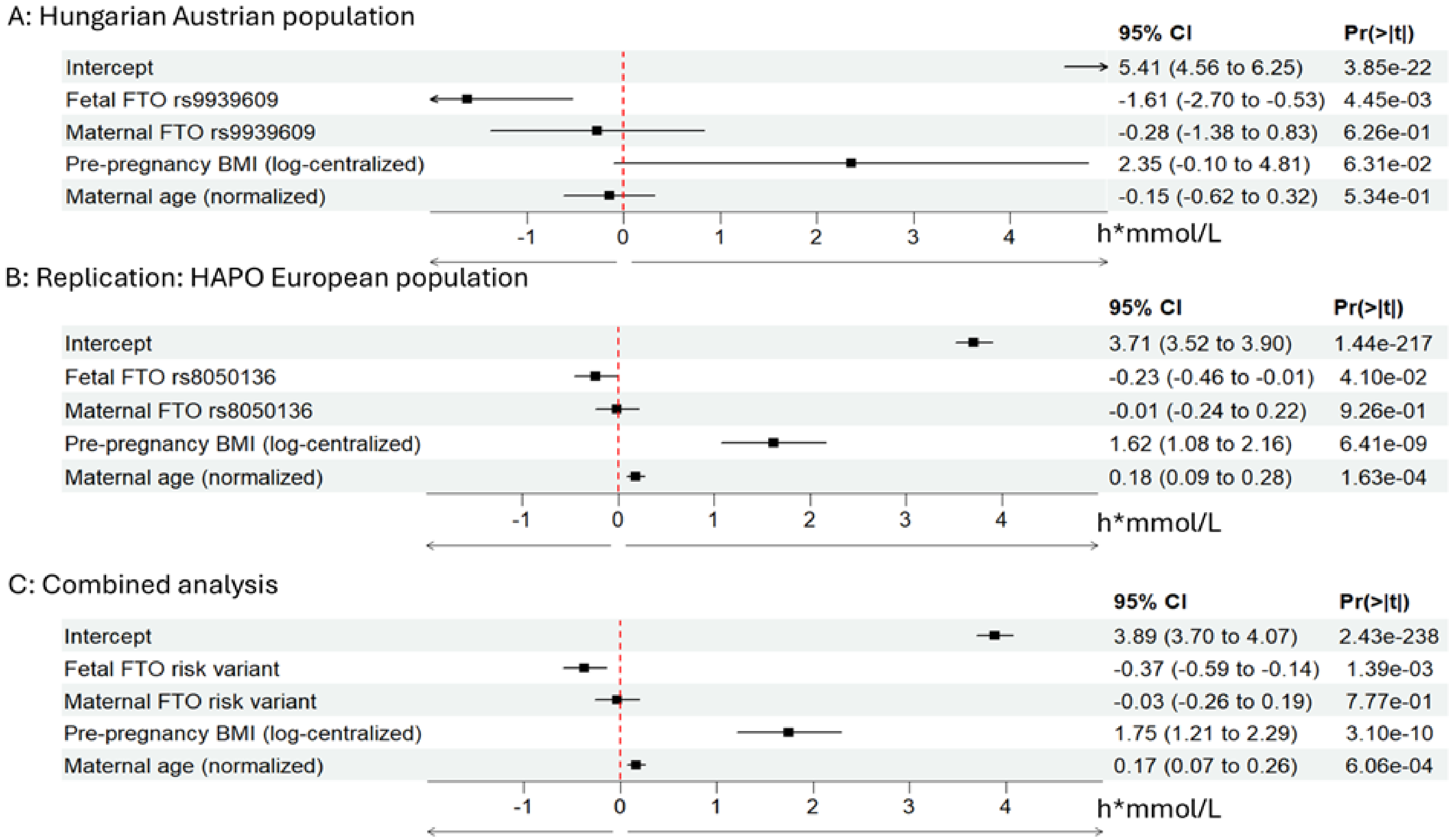

Supplementary Figure 5. Fetal and maternal *FTO* genetic effect on AUC(gluc)INC during OGTT in Hungarian-Austrian (A), HAPO-EUR (B) and in the combined study populations (C)

AUC(gluc)INC, incremental area under glucose curve**;** HAPO-EUR, Hyperglycemia Adverse Pregnancy Outcome study subpopulation of European ethnic origin

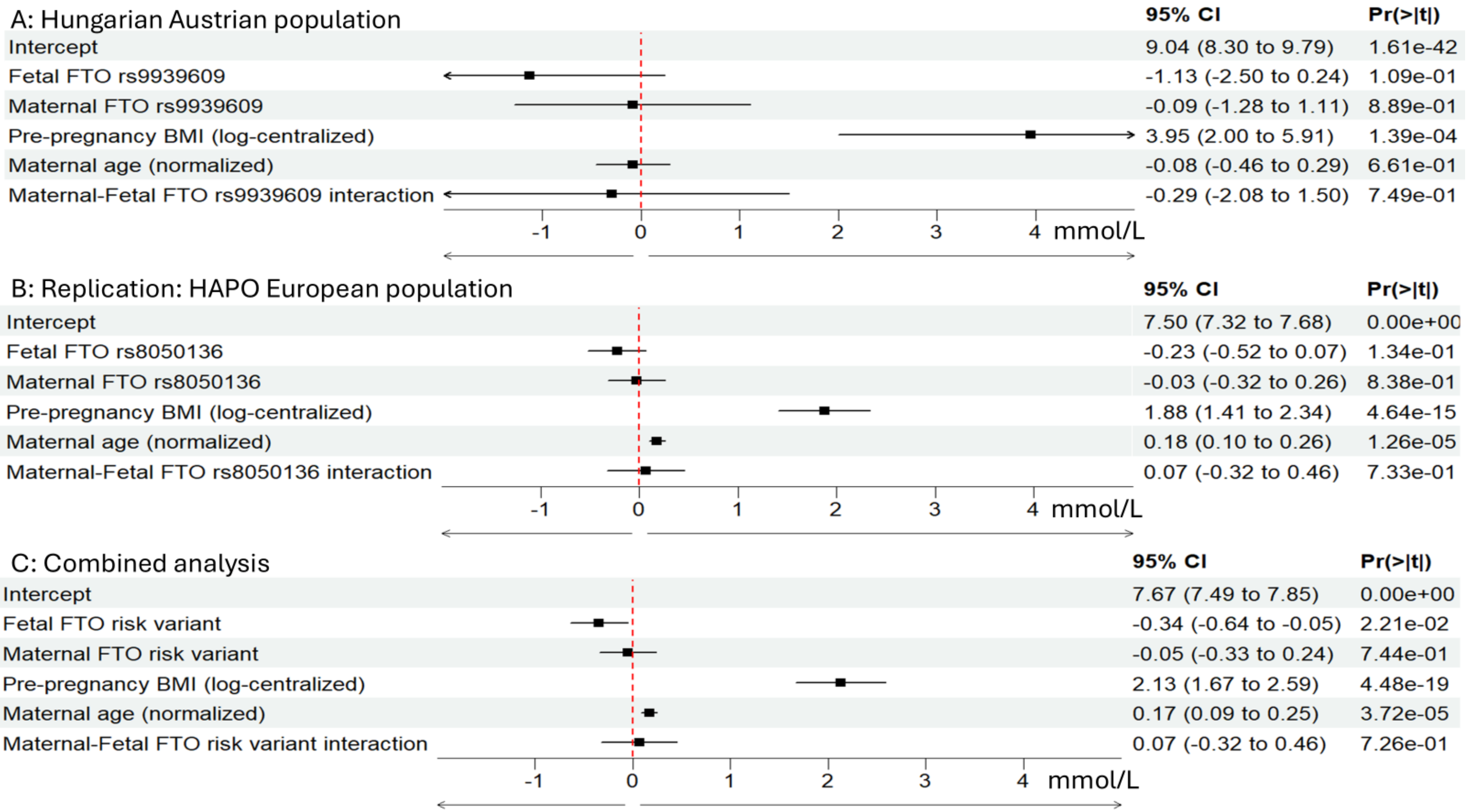

Supplementary Figure 6. Interaction analysis of fetal and maternal *FTO* genetic effects on 60' plasma glucose values (during OGTT) in Hungarian-Austrian (A), HAPO (B) and in the combined population (C)

HAPO-EUR, Hyperglycemia Adverse Pregnancy Outcome study subpopulation of European ethnic origin

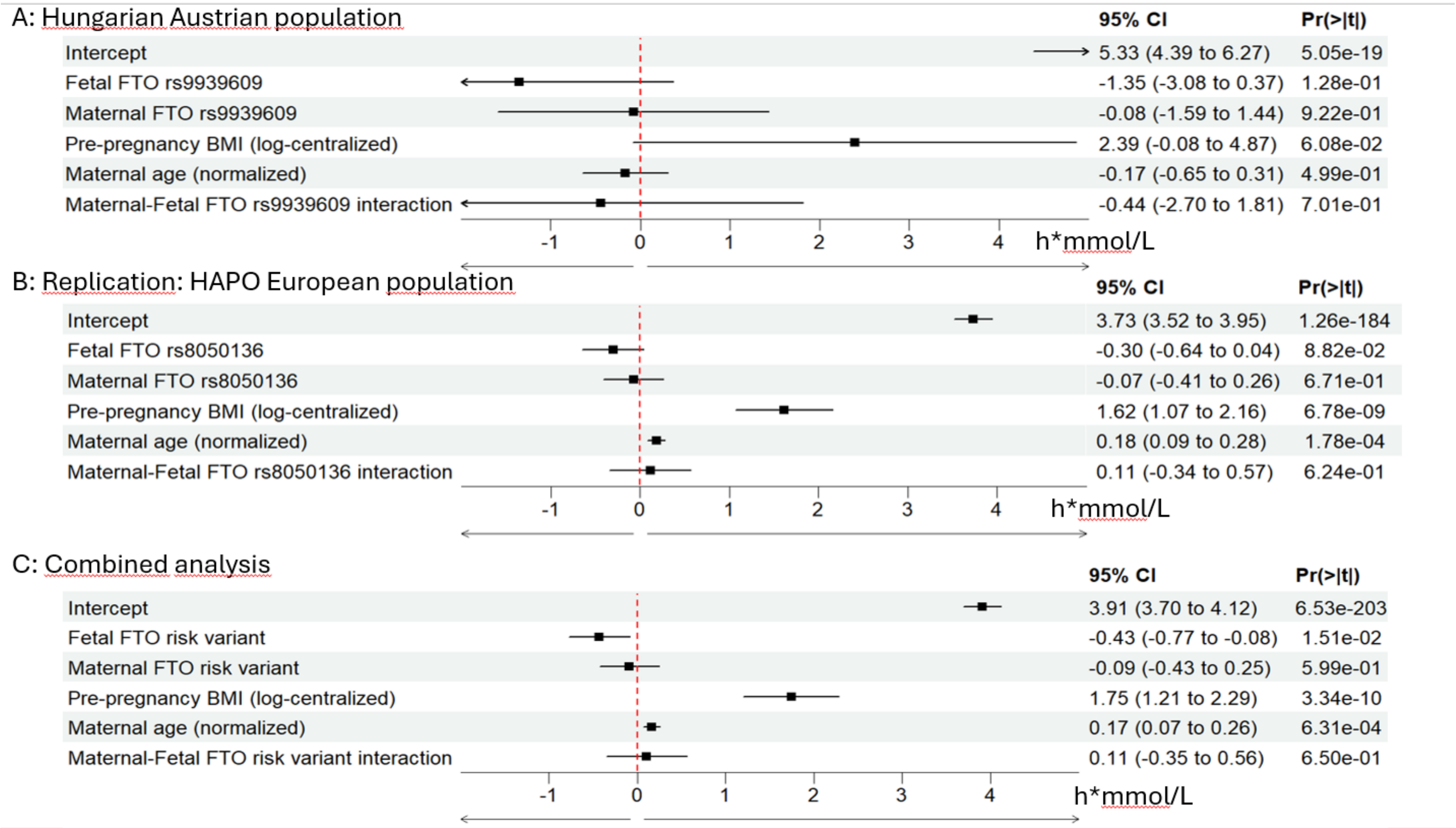

Supplementary Figure 7. Interaction analysis of fetal and maternal *FTO* genetic effects on AUC(gluc)INC during OGTT in Hungarian-Austrian (A), HAPO_EUR (B) and in the combined study populations (C)

AUC(gluc)INC, incremental area under glucose curve**;** HAPO-EUR, Hyperglycemia Adverse Pregnancy Outcome study subpopulation of European ethnic origin

Panel A: ***FTO*** **rs9939609 (Hungarian-Austrian study population)**	**Minor Allele Frequency**	**Number of cases**				**Observed genotype distribution**			**Expected genotype distribution (HWE)**			**Chi-square test**	
		**Homozygous for Major Allele *(CC)***	**Heterozygous *(CA)***	**Homozygous for Minor Allele *(AA)***	**All**	***CC***	***CA***	***AA***	***CC***	***CA***	***AA***	**X^2**	**p-value**
**Neonate HUN-AUS entire study population**	0.427	236	342	132	710	0.332	0.482	0.186	0.329	0.489	0.182	0.17	0.918
**Mother HUN-AUS entire study population**	0.411	241	291	124	656	0.367	0.444	0.189	0.347	0.484	0.169	4.59	0.101
**Neonate HUN-AUS study population analyzed**	0.328	60	48	17	125	0.480	0.384	0.136	0.452	0.441	0.108	2.08	0.354
**Mother HUN-AUS study population analyzed**	0.375	43	44	17	104	0.413	0.423	0.163	0.391	0.469	0.141	0.99	0.610

Panel B: ***FTO*** **rs8050136 (HAPO-EUR study population)**	**Minor Allele Frequency**	**Number of cases**				**Observed genotype distribution**			**Expected genotype distribution (HWE)**			**Chi-square test**		**Chi-square test between study populations**	
		**Homozygous for Major Allele (*TT*)**	**Heterozygous (*TA*)**	**Homozygous for Minor Allele (*AA*)**	**All**	***TT***	***TA***	***AA***	***TT***	***TA***	***AA***	**X^2**	**p-value**	**X^2**	**p-value**
**Neonate HAPO entire study population**	0.403	494	653	227	1374	0.360	0.475	0.165	0.357	0.481	0.162	0.204	0.903	2.190	0.335
**Mother HAPO entire study population**	0.404	465	628	215	1308	0.356	0.480	0.164	0.355	0.482	0.164	0.015	0.993	2.955	0.228
**Neonate HAPO study population analyzed**	0.403	494	653	227	1374	0.360	0.475	0.165	0.357	0.481	0.162	0.204	0.903	7.140	0.028
**Mother HAPO study population analyzed**	0.404	465	628	215	1308	0.356	0.480	0.164	0.355	0.482	0.164	0.015	0.993	1.559	0.459

Supplementary Table 1. Case numbers and genotype distributions of the *FTO* risk gene variants in the primary (Hungarian-Austrian, panel "A") and replication (HAPO-EUR) study (panel "B") populations

HAPO, Hyperglycemia Adverse Pregnancy Outcome study; HAPO-EUR, Hyperglycemia Adverse Pregnancy Outcome study subpopulation of European ethnic origin; HUN-AUS; Hungarian-Austrian; $\chi 2$, Chi-squared; HWE, Hardy-Weinberg Equilibrium
